# Antimicrobial resistance of clinical bacterial isolates in China: current status and trends

**DOI:** 10.1093/jacamr/dlae052

**Published:** 2024-03-28

**Authors:** Xiaohua Qin, Li Ding, Min Hao, Pei Li, Fupin Hu, Minggui Wang

**Affiliations:** Institute of Antibiotics, Huashan Hospital, Fudan University, Shanghai, China; Key Laboratory of Clinical Pharmacology of Antibiotics, National Heath Commission of the People’s Republic of China, Shanghai, China; Institute of Antibiotics, Huashan Hospital, Fudan University, Shanghai, China; Key Laboratory of Clinical Pharmacology of Antibiotics, National Heath Commission of the People’s Republic of China, Shanghai, China; Institute of Antibiotics, Huashan Hospital, Fudan University, Shanghai, China; Key Laboratory of Clinical Pharmacology of Antibiotics, National Heath Commission of the People’s Republic of China, Shanghai, China; Institute of Antibiotics, Huashan Hospital, Fudan University, Shanghai, China; Key Laboratory of Clinical Pharmacology of Antibiotics, National Heath Commission of the People’s Republic of China, Shanghai, China; Institute of Antibiotics, Huashan Hospital, Fudan University, Shanghai, China; Key Laboratory of Clinical Pharmacology of Antibiotics, National Heath Commission of the People’s Republic of China, Shanghai, China; Institute of Antibiotics, Huashan Hospital, Fudan University, Shanghai, China; Key Laboratory of Clinical Pharmacology of Antibiotics, National Heath Commission of the People’s Republic of China, Shanghai, China

## Abstract

Antimicrobial resistance surveillance systems have been established in China. Two representative national surveillance networks are the China Antimicrobial Surveillance Network (CHINET) and China Antimicrobial Resistance Surveillance System (CARSS), both of which were established in 2005. For all clinical isolates collected in both of these surveillance networks, the ratio of Gram-negative bacilli to Gram-positive cocci was approximately 7:3 during the past 18 years. Generally, Gram-negative bacilli have a higher antimicrobial resistance profile in China. The prevalence of ESBLs in *Escherichia coli* is as high as approximately 50%. *Acinetobacter baumannii*-*calcoaceticus* complex (ABC) has a high antimicrobial resistance profile, with a carbapenem resistance rate of approximately 66%. However, the prevalence of carbapenem-resistant ABC has also shown a decreasing trend from 2018 to 2022. The prevalence of vancomycin-resistant *Enterococcus* was low, and the prevalence of MRSA and carbapenem-resistant *Pseudomonas aeruginosa* showed decreasing trends from 2005 to 2022. CHINET surveillance data demonstrated that the prevalence of carbapenem-resistant *Klebsiella pneumoniae* showed a remarkable increasing trend from 2.9% (imipenem resistance) in 2005 to 25.0% in 2018, and then slightly decreased to 22.6% in 2022. The decreasing trends may reflect the antimicrobial stewardship efforts in China: a professional consensus on the rational clinical use of carbapenems was issued by the National Health Commission of China and was well implemented nationally; after that, the clinical use of carbapenems decreased slightly in China.

## Introduction

Antimicrobial resistance (AMR) has become a major global threat not only to public health but also to the social economy.^1^ In 2022, 13 ministries of China led by the National Health Commission jointly updated the National Action Plan for AMR Containment (2022–25). Surveillance of bacterial resistance, which is important for the clinical empirical and targeted treatment of infection, is regarded as one of the primary tasks in this national action plan for solving the AMR problem. In this review, the current status and trends of AMR from 2005 to 2022 among common clinical isolates in China are presented, and the mechanisms of resistance for some AMR isolates are reviewed. Most of the resistance data were obtained from the two national bacterial AMR surveillance networks in China: China Antimicrobial Surveillance Network (CHINET; https://www.chinets.com) and China Antimicrobial Resistance Surveillance System (CARSS; https://www.carss.cn).

## AMR surveillance system in China

AMR surveillance systems have been well established in China, including at hospital, province and national levels. Hospital-level AMR surveillance is required as the main task of antimicrobial stewardship measures, as local AMR data are crucial for rational, empirical administration of antibiotics, especially in hospital-acquired infections. The surveillance data were required to be fed back to clinicians via the hospital intranet and booklet distribution every quarter or half of the year.^2^

Province-level AMR surveillance has been established over the past 35 years. Each province-level network includes more than 10 hospitals from all regions in each province and releases an annual report of AMR data. The Shanghai Bacterial Resistance Surveillance Network was established in 1988 with the help of the WHO Western Pacific Region Office.^2^

Two national surveillance networks for bacterial AMR, CHINET and CARSS, were established in 2005. CHINET was launched by Huashan Hospital, affiliated with Fudan University, and it expanded to 73 hospitals across 29 provinces, municipalities and autonomous regions by 2022, including 53 general hospitals and 20 children’s hospitals, of which 57 were tertiary hospitals and 16 were secondary hospitals.^3^ The CHINET surveillance system is dedicated to helping clinicians better understand the current status and trends of AMR and correct inappropriate prescription behaviours, thus assisting experts in formulating relevant guidelines and consensus in China and other Asian countries. More than 20 guidelines or expert consensus statements have been published with reference to the CHINET data.^4^ ‘CHINET Cloud,’ an open-access online information network station of AMR, releases the latest surveillance data annually (semi-annually since 2022). Patients, physicians and researchers can browse heat maps, bar charts and trend charts of various bacteria in different years. Thousands of users are registered on the CHINET Cloud. The CARSS is organized by the Expert Committee on Rational Use of Drugs of the National Health Commission of the People’s Republic of China. By the year 2021, 1997 hospitals from 31 provinces and autonomous regions had participated, including 1411 tertiary and 586 secondary hospitals.^5^ In this review, the comparison of AMR status among different provinces for each of the major clinical isolates was based on the CARSS data.

Only one clinical isolate of the same species was included per patient per year based on their personal identification code to avoid isolates duplication. According to a CHINET uniform protocol, antimicrobial susceptibility testing (AST) was performed using automated systems in every local lab separately, supplemented by a disc diffusion method for some antimicrobial agents not available in automated systems. Quality control and test results were interpreted according to CLSI breakpoints for all agents tested, except for tigecycline and colistin. Tigecycline MICs, for which CLSI criteria were not available, were interpreted using US FDA MIC breakpoints. Colistin MICs were interpreted using the MIC interpretive breakpoints for colistin. The methods were consistent in all participating hospitals, which were verified every year.^6^ The CARSS protocol of AST was the same with CHINET.

## Distribution of clinical isolates

**Figure 1.**
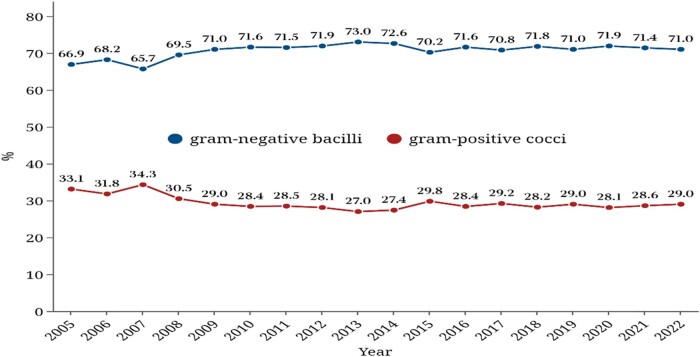
Percentages of Gram-positive cocci and Gram-negative bacilli of clinical isolates in China (CHINET data).

According to CHINET surveillance data from 2005 to 2022, the ratio of Gram-negative bacterial isolates to Gram-positive cocci remained high: 70% versus 30% (Figure 1).^3^ Similarly, the ratio was 71.5% versus 28.5% in the CARSS surveillance of 2022.^5^

**Figure 2.**
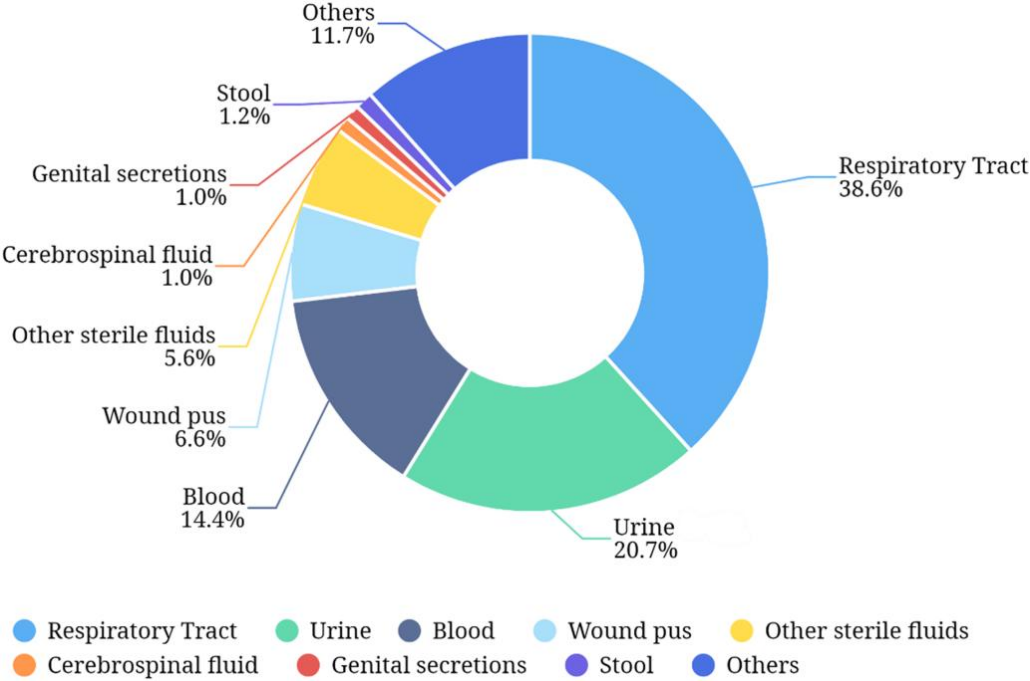
Specimen source distribution of the 339 513 clinical isolates in CHINET 2022.

**Figure 3.**
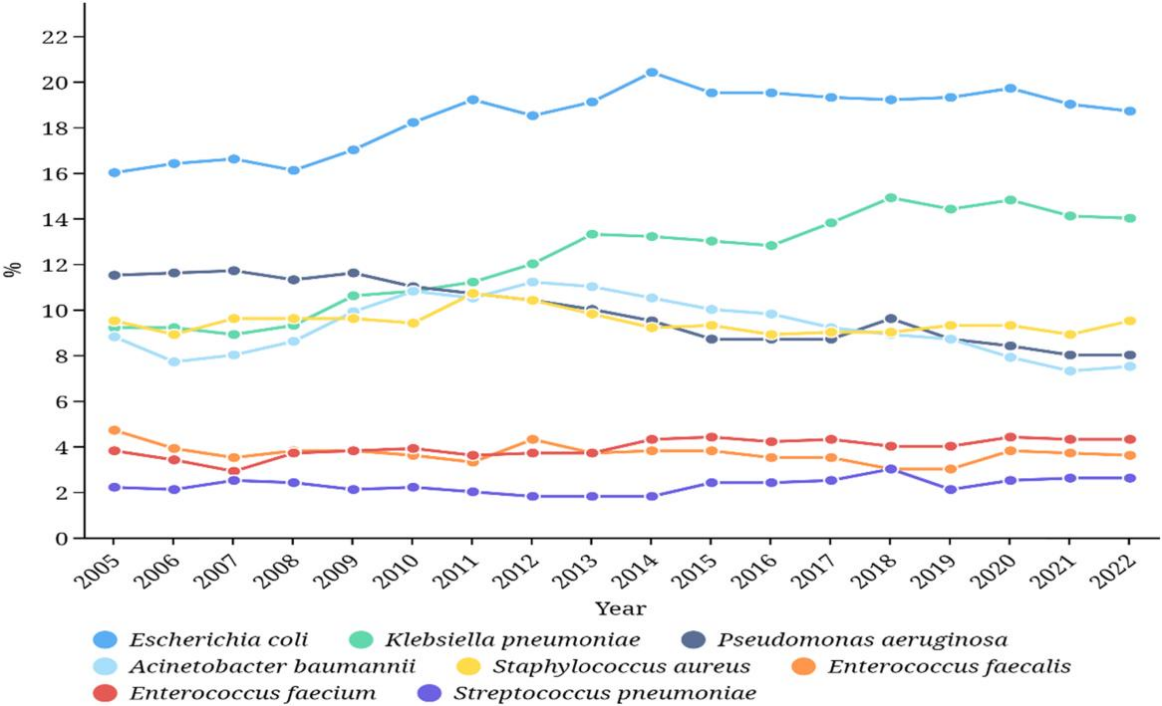
Percentages of the top eight clinical isolates (CHINET data).

According to CHINET 2022, most isolates were cultured from the lower respiratory tract (38.6%), followed by urine (20.7%), blood (14.4%) and wound pus (6.6%) (Figure 2).^3^ Likewise, of the clinical isolates included in CARSS 2022, 38.3% were isolated from the respiratory tract, 21.1% from urine and 8.5% from blood.^5^ The eight most frequently cultured species in CHINET 2022 were *Escherichia coli* (18.7%), *Klebsiella pneumoniae* (14.0%), *Staphylococcus aureus* (9.5%), *Pseudomonas aeruginosa* (8.0%), *Acinetobacter baumannii*-*calcoaceticus* complex (ABC) (7.5%), *Enterococcus faecium* (4.8%), *Enterococcus faecalis* (3.4%) and *Streptococcus pneumoniae* (2.6%) (Figure 3).^3^

*E. coli* was always ranked as the most common species from 2005 to 2022, representing 16.0%–20.4% of all isolate species, while the percentage of *K. pneumoniae* showed a significant increasing trend, from 9.5% in 2005 to 14% in 2022. By contrast, *P. aeruginosa* presented a decreasing trend from 11.5% in 2005 to 8.0% in 2022. The prevalence of ABC increased from 9.4% in 2005 to 11.0% in 2012 and, after that, decreased to 7.5% in 2022. For Gram-positive cocci, there was little change in the ranking, as *S. aureus* fluctuated around 10%, *E. faecium* and *E. faecalis* around 5% and 4%, respectively, and *S. pneumoniae* was 2.5% in sequence according to CHINET 2005–22 (Figure 3).^3^ In CARSS 2022, the top four Gram-negative bacilli were also *E. coli*, *K. pneumoniae*, *P. aeruginosa* and ABC, whereas the top four Gram-positive cocci were *S. aureus*, *E. faecium*, *E. faecalis* and *S. epidermidis* in order.^5^

## AMR prevalence and trends in Gram-negative bacilli

### E. coli

**Figure 4.**
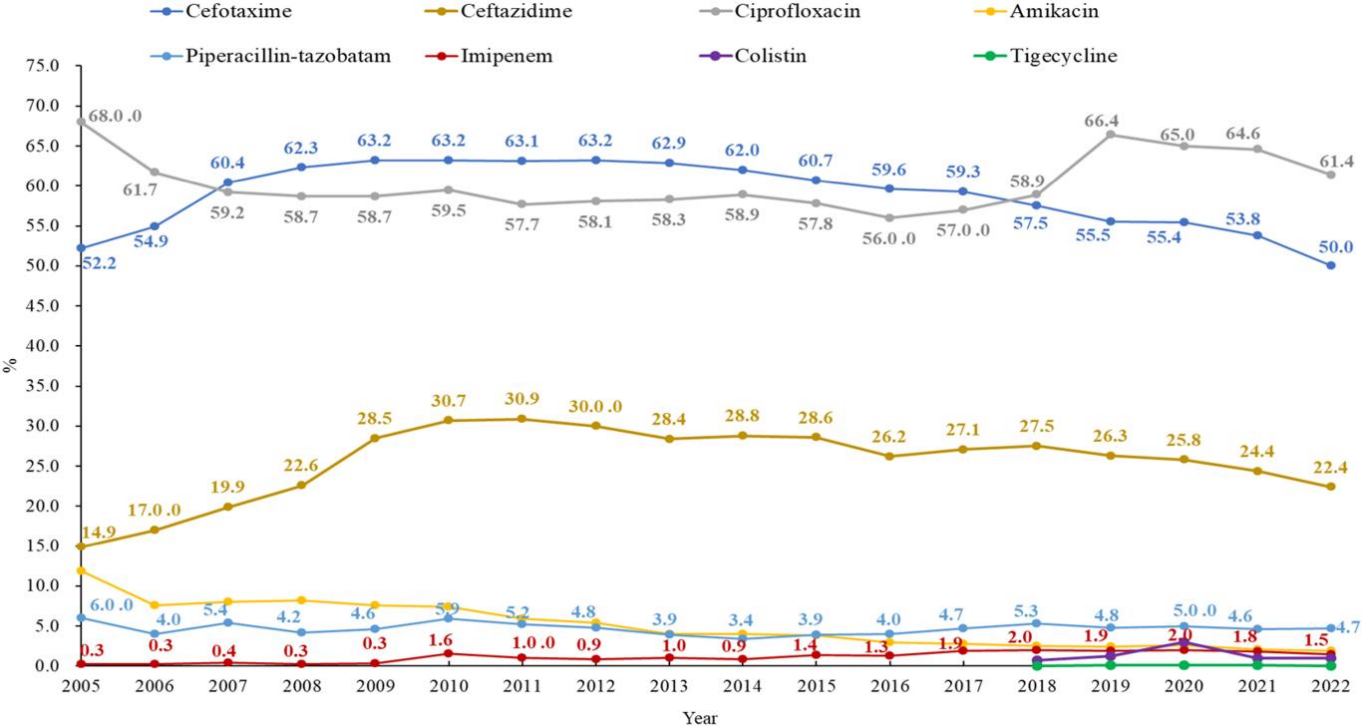
Resistance profile of *E. coli* for six commonly used antimicrobials (CHINET data). The CLSI breakpoints of fluoroquinolones for Enterobacterales changed in January 2019

According to CHINET, cefotaxime resistance rates in *E. coli* increased from 52.2% in 2005 to 63.2% in 2012 and then decreased gradually to 50% in 2022, while ceftazidime resistance rates peaked at 30.9% in 2011, and then decreased steadily to 22.4% in 2022. The main genotype of ESBLs in China is CTX-M. Cefotaxime or ceftriaxone resistance may be considered a marker of ESBL-producing isolates for carbapenem-susceptible strains, as the *bla*_CTX-M_*-*positive isolates were all resistant to cefotaxime or ceftriaxone.^7^ Ciprofloxacin resistance rates decreased from 68% in 2005 to 56% in 2016. Still, they escalated to 66.4% in 2019, which was due to the CLSI changing the breakpoints of fluoroquinolones for Enterobacterales in January 2019. According to the breakpoint of ciprofloxacin resistance, the MIC of *E. coli* changed from ≥4 to ≥1 mg/L. Amikacin resistance rates decreased smoothly from 11.9% in 2005 to 2.2% in 2022, while gentamicin resistance rates were above 30% in the last 5 years, according to the CHINET network. Resistance rates to piperacillin/tazobactam and imipenem were low in *E. coli*, ranging from 4%–6% and 0.7%–2.0%, respectively (Figure 4). The major mechanism of carbapenem resistance in carbapenem-resistant *E. coli* (CREC) is the production of MBL, predominantly NDM-1 and NDM-5. Other carbapenemases detected in CREC include KPCs, IMP and OXA, which are less common in *E. coli.*^8,9^ The resistance rates of colistin and tigecycline in *E. coli* have fluctuated around 1.0% and 0.1%, respectively, over the recent 5 years.^6^

### K. pneumoniae

**Figure 5.**
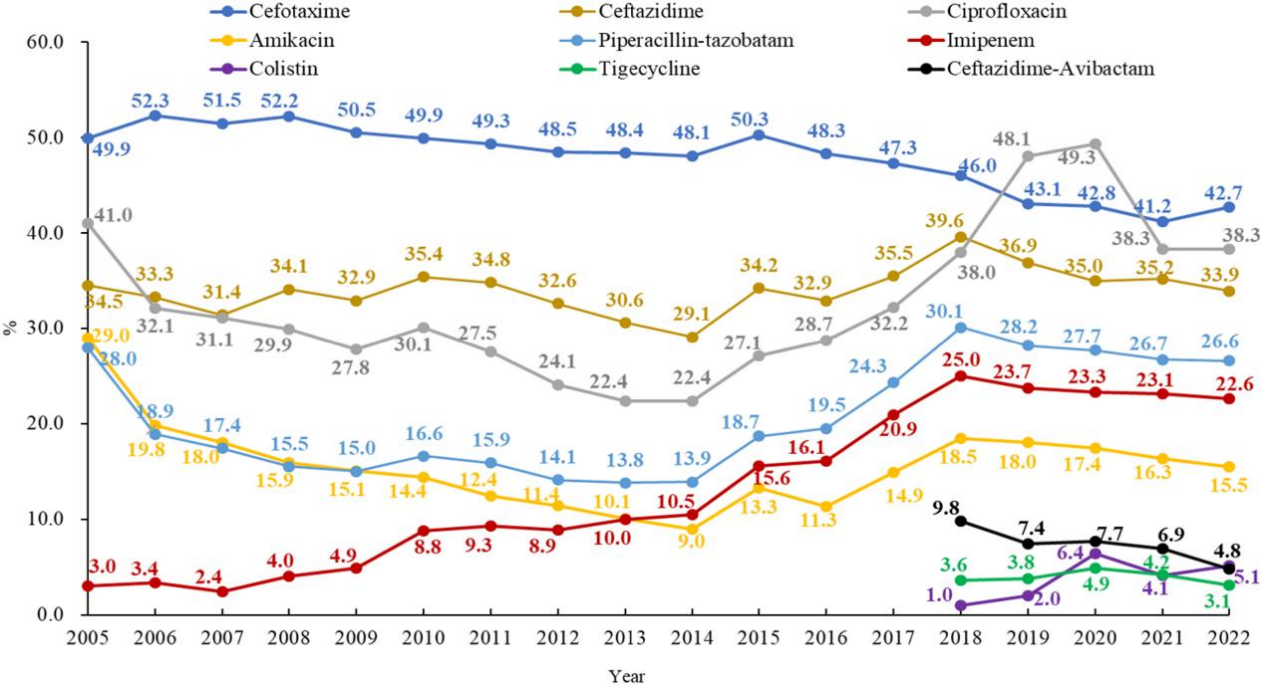
Resistance profile of *K. pneumoniae* for six commonly used antimicrobials (CHINET). The CLSI breakpoints of fluoroquinolones for Enterobacterales changed in January 2019.

**Figure 6.**
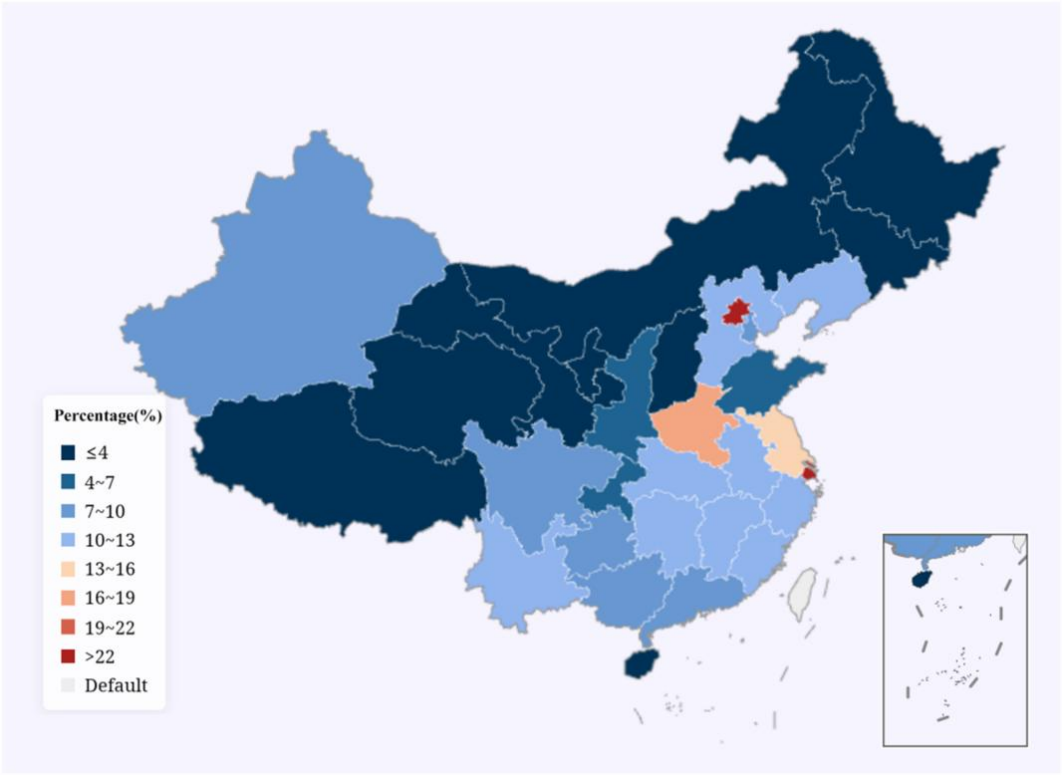
Prevalence of CRKP among different provinces in China in 2022 (CARSS).

The cefotaxime resistance rate in *K. pneumoniae* decreased slightly from 52.3% in 2006 to 42.7% in 2022 and the piperacillin/tazobactam resistance rate peaked at 30.1% in 2018 but decreased slightly to 26.6% in 2022. The prevalence of carbapenem-resistant *K. pneumoniae* (CRKP) increased from 2.9% (imipenem resistance) in 2005 to 25.0% in 2018, while it decreased slightly to 22.6% in 2022 (Figure 5).^3^ This primarily was attributed to the consensus issued by the National Health Commission on the rational application of carbapenems to strengthen the management of the clinical use of carbapenems in 2018. The prevalence of CRKP ranged from 0.8% to 28.1% in different provinces, with an average rate of 10% (showing an increase from 7.4% in 2016) in the CARSS surveillance in 2022 (Figure 6).^5^ The production of carbapenemases, especially KPCs, is the main mechanism of carbapenem resistance in CRKP, and the bla_KPC-2_ genotype accounts for more than 70%.^9^ The ceftazidime/avibactam resistance rate in *K. pneumoniae* isolates has been as low as 6% in the last two years.^6^ The most common mechanism of carbapenem resistance in *K. pneumoniae* collected from children is KPC-2, but the prevalence of NDM, especially NDM-1, is higher than in adults in China.^10,11^ The dominant clone of CRKP is ST11, which usually carries multiple resistance genes via plasmids and causes nosocomial infection outbreaks.^12^ The spread of ST11 may have contributed to the increasing trend of multiple and even extensive drug resistance in *K. pneumoniae* since 2015.^2^ The colistin resistance rates increased from 1.1% in 2018 to 5.0% in 2022, and tigecycline resistance fluctuated between 2.5% and 5.2% in the last 5 years (Figure 5).^3^

### P. aeruginosa

**Figure 7.**
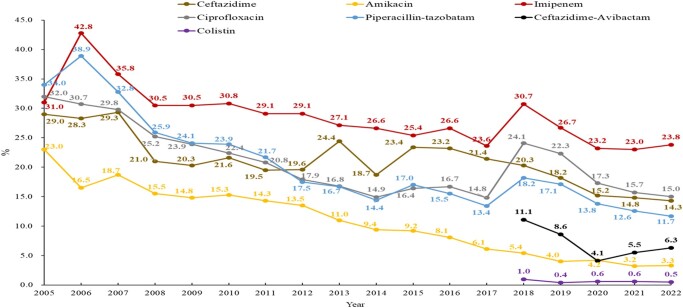
Resistance profile of *P. aeruginosa* for five commonly used antimicrobials (CHINET data).

*P. aeruginosa* had a relatively low resistance profile and a trend of decreasing resistance to the commonly used antimicrobials. The amikacin resistance rates dropped significantly from 23.0% in 2005 to 3.3% in 2022. The ceftazidime, piperacillin/tazobactam and ciprofloxacin resistance rates also dropped to 14.3%, 11.7% and 15.0%, respectively, in 2022.^3^ Carbapenem-resistant *P. aeruginosa* (CRPA) ranged from 7.6% in the Ningxia autonomous region to 26% in Shanghai, with an average rate of 16.6% in CARSS in 2022,^5^ while the prevalence of CRPA was 23.8% in CHINET in 2022.^3^ The most common mechanisms of CRPA are overexpression of the efflux pump MexAB-OprM and inactivation of the outer membrane protein.^13^ Of the CRPA isolates, 32% had a carbapenemase gene in China, with the most common type being *bla*_KPC-2_ (23%–40.4% among CRPA strains), followed by *bla*_VIM-2_.^14,15^ Among the KPC-producing *P. aeruginosa* isolates, 50.3% (76/151) were resistant to ceftazidime/avibactam.^15^ Resistance to ceftazidime/avibactam significantly decreased from 11.1% to 6.3%, and the resistance rate of colistin fluctuated around 0.5% in *P. aeruginosa*, according to CHINET 2018–22 (Figure 7).^3^

### ABC

**Figure 8.**
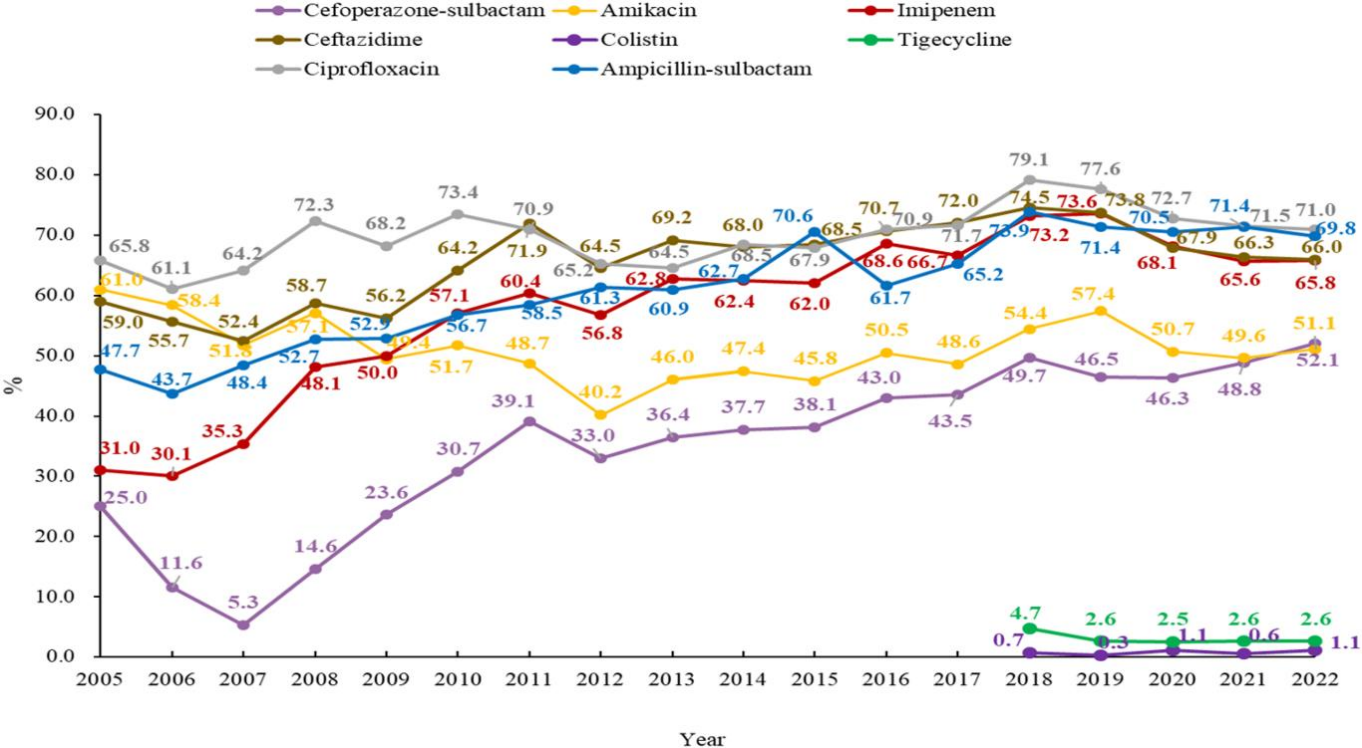
Resistance profile of ABC for five commonly used antimicrobials (CHINET data).

Generally, the resistance rates of ABC against all the commonly used antimicrobials were very high and showed an increasing trend, except for amikacin, according to CHINET 2005–22 (Figure 8). Resistance to ampicillin/sulbactam, cefoperazone/sulbactam and amikacin was 69.8%, 52.1% and 51.1%, respectively, in CHINET 2022.^3^ Imipenem resistance rates increased from 31% in 2005 to 74.5% in 2018 and decreased slightly to 65.8% in 2022. Carbapenem-resistant ABC (CRABC) varied among provinces, with the lowest prevalence of 23.3% in Qinghai Province to the highest of 71% in Henan Province, with an average of 53.4% according to the results of CARSS in 2022.^5^ Clonal group 2 isolates producing acquired oxacillinases (Ambler class D β-lactamases), especially OXA-23 carbapenemases, account for a large proportion of CRABC cases worldwide, while 99% of CRABC produce OXA-23 in China. Other carbapenemase resistance genes, such as *bla*_OXA-24_, *bla*_NDM-237_ and *bla*_NDM-58_, have also been observed worldwide.^16^ Polymyxin and tigecycline resistance rates fluctuate around 1% and 2%, respectively.^3^

## AMR prevalence and trends in Gram-positive cocci

### S. aureus

**Figure 9.**
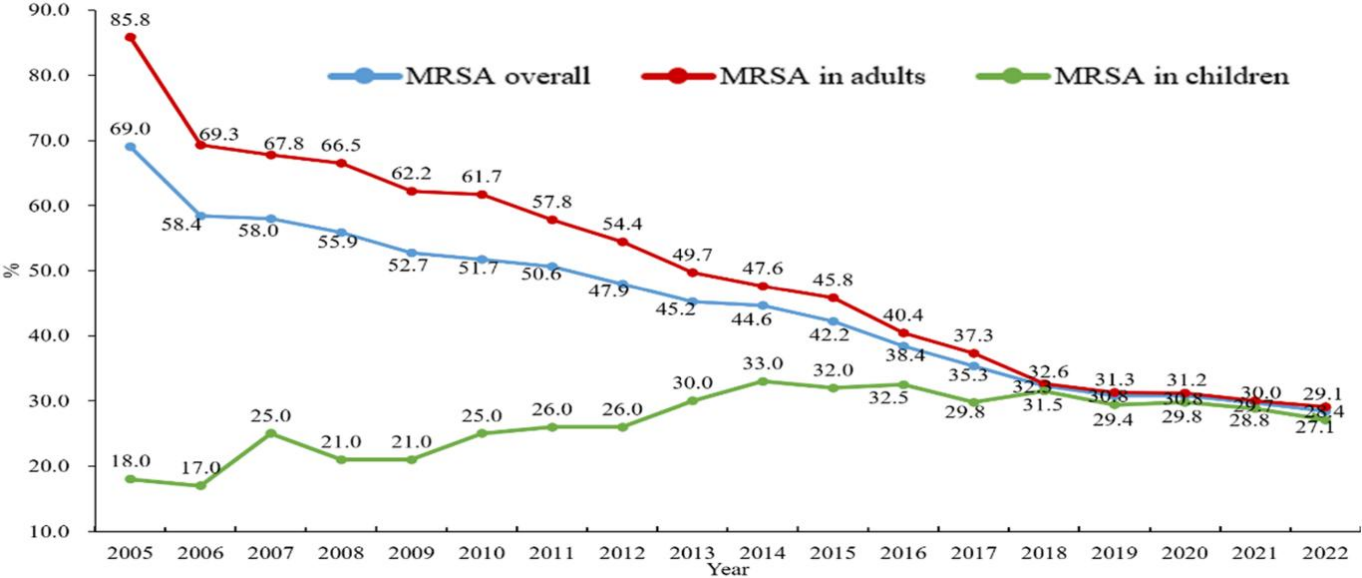
Prevalence of MRSA overall and in adults (≥18 years old) and children (<18 years) CHINET 2005–22.

**Figure 10.**
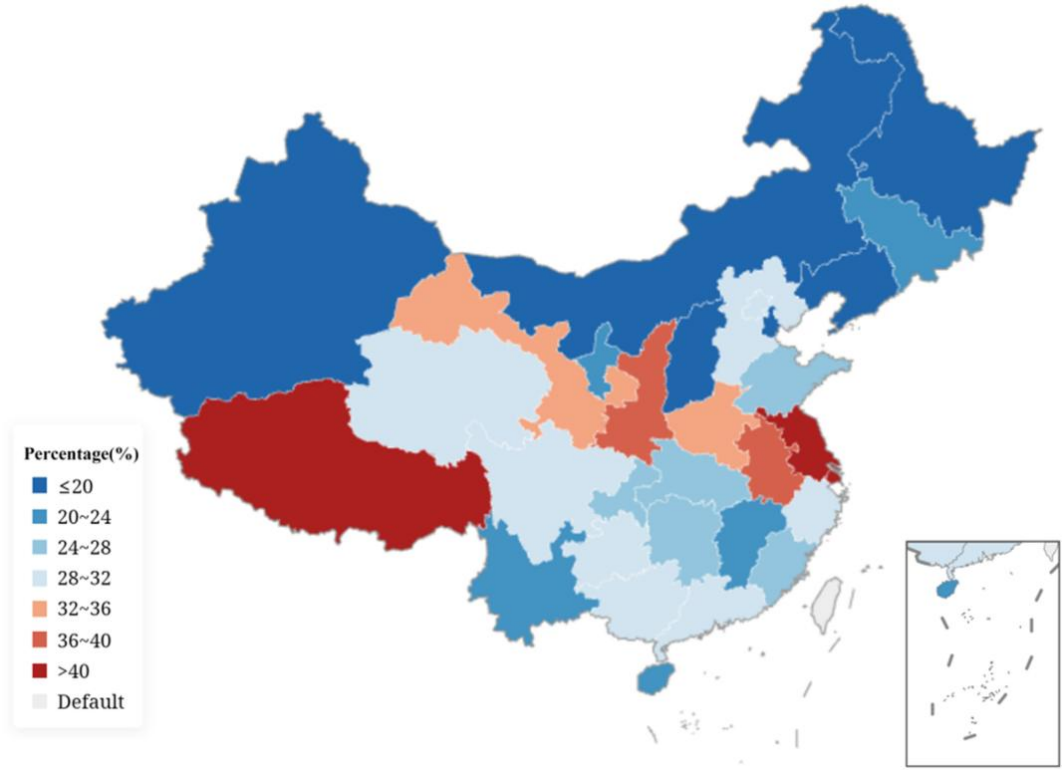
Prevalence of MRSA among different provinces in China among different provinces in China in 2022 (CARSS).

The prevalence of MRSA has shown a remarkable decreasing trend from 69.0% in 2005 to 28.7% in 2022, according to CHINET data. The prevalence of MRSA in adults aged ≥18 years decreased from 85.8% in 2005 to 29.1% in 2022, whereas that in children aged <18 years increased from 18.0% to 27.1% during the same period (Figure 9).^3^ No MRSA isolate was resistant to vancomycin or linezolid, and resistance to sulfamethoxazole/trimethoprim was as low as 6.6%, while resistance to clindamycin was as high as 53% in CHINET 2022.^3^ The decreasing prevalence of MRSA is related to the effective implementation of nosocomial infection control measures, such as hand hygiene and antimicrobial stewardship measures in clinical practice in China. ST239 has historically been the dominant lineage in hospital infections in mainland China, but since 2013 community-associated strains including ST59 have largely been replacing the previously dominant healthcare-associated ST239.^17^ It is not clear whether the clone replacement is related to the decreasing MRSA prevalence. However, the significant increase in MRSA prevalence in children is a cause for concern. The increasing trend in children may be related to the increasing number of ICU beds in children’s hospitals or departments that usually have higher bacterial resistance rates, including the prevalence of MRSA; limited choices of antimicrobials for treatment compared with adults may also be related to the increasing trend of MRSA prevalence. Other factors including shorter bed-to-bed distance and more frequent invasive operations such as aspiration of sputum may also be related to the increasing MRSA prevalence in children. Further studies focused on the increasing prevalence of MRSA in children are required in the future. The average MRSA prevalence among different provinces was 28.9%, ranging from 16% in Liaoning Province to 44% in the Xizang Autonomous Region, according to CARSS surveillance in 2022 (Figure 10).^4^

### Enterococci

**Figure 11.**
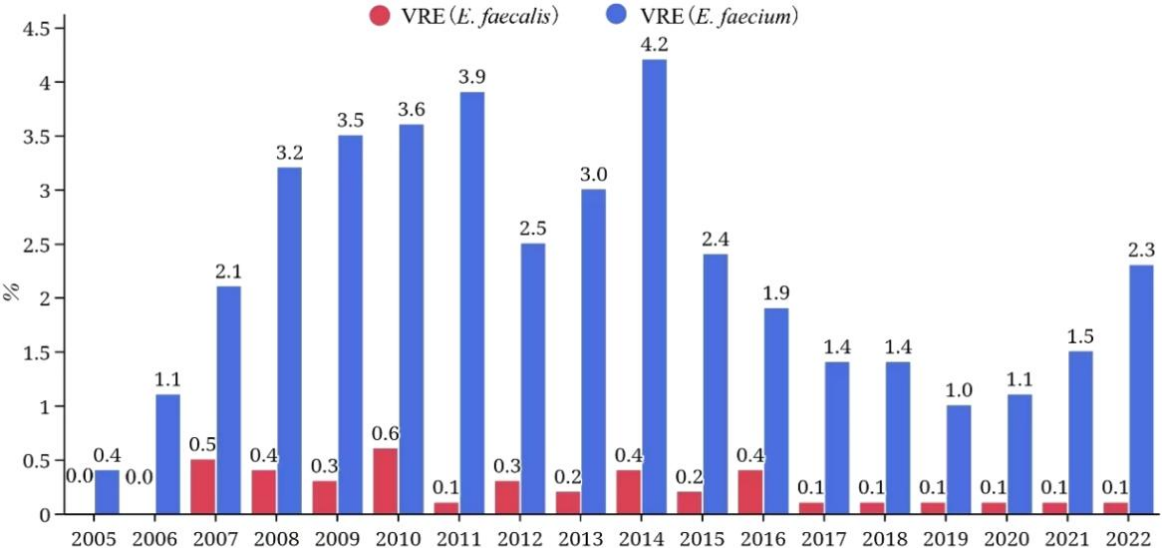
Prevalence of vancomycin-resistant *Enterococcus faecalis* and *Enterococcus faecium* CHINET 2005–22.

The prevalence of VRE was relatively low in China: <5% for *E. faecium* and <1% for *E. faecalis* in the CHINET surveillance from 2005 to 2022 (Figure 11).^3^ In the CARSS surveillance, the mean VRE prevalence was 1.7% for *E. faecium*, ranging from 0% to 11.7% across different provinces.^5^ The prevalence of VRE is much lower than that reported in many other countries around the world. The low VRE prevalence may be partially related to the infrequent use of oral vancomycin preparations, which are not available in China. IV preparations are administered orally to treat *Clostridioides difficile* infections if needed. Avoparcin has never been approved for use in animals in China. Enterococci become resistant to vancomycin by acquiring genes that influence cell wall formation.^18^ The genotypes encoding resistance to glycopeptides include *vanA-G*, *vanL* and *vanM*, and the predominant type prevalent in China and worldwide is *vanA.*^19^ The *vanM* gene was initially identified in a vancomycin-resistant *E. faecium* clinical isolate from a hospital in Shanghai, China, in 2006 and has been predominant in Shanghai since 2011. *vanM* has been detected in many countries, including Singapore, Japan and Canada.^20^

### S. pneumoniae

In 2017, the prevalence of penicillin-non-susceptible *S. pneumoniae* (PNSP), including penicillin-resistant *S. pneumoniae* and penicillin-intermediate *S. pneumoniae*, was higher in children than in adults (13.5% versus 8.2%). However, the gap has been narrowed since, as the prevalence of PNSP has reduced to 5.5% in children, while it had decreased to 4.6% in adults in the CHINET surveillance 2022.^3^ More than 90% of *S. pneumoniae* isolates were resistant to erythromycin or clindamycin in both children and adults. Of the *S. pneumoniae* isolates, 4.1% were resistant to levofloxacin, and 2.1% to moxifloxacin in adults, and even lower resistance rates (less than 0.5%) were found in children.^3^ Mutations in PBPs are the main mechanism underlying penicillin resistance in *S. pneumoniae*. In China, the most prevalent serotypes were 19A (20.9%) and 23F (20.3%) in children and 3 (21.7%) and 19F (11.8%) in adults.^21^ The main serotypes in CSF isolates were 23F and 19F.^22^

In general, Gram-negative bacterial clinical isolates are much more common than Gram-positive cocci, with a ratio of approximately 7:3, and Gram-negative bacilli have higher AMR profiles; therefore, the AMR of Gram-negative bacilli is a dominant problem in China. The prevalence of ESBLs in *E. coli* remains as high as 50%, and the prevalence of CRKP is currently approximately 25%. *A. baumannii* has the highest AMR profile, with a CRABC prevalence of approximately 66%. The prevalence of MRSA and AMR in *P. aeruginosa* showed a decreasing trend from 2005 to 2022, and the prevalence of VRE was low.

The present review was based on the data of CHINET and CRASS over the past 18 years but still had some limitations. The members of both surveillance networks were increasing during the surveillance period, which may somewhat affect the comparability of the annual AMR data. Although the AST followed the uniform protocol, the testing was carried out separately at local labs of the participating hospitals. There was a lack of statistical analysis to clarify the real significance of AMR trends. In the future, it is important to have close monitoring on changing trends of carbapenem-resistant organism surveillance including CRKP, CRABC and CRPA. The higher MRSA prevalence in children caught wide attention; studies to identify the potential causes would be helpful to control the increasing trends of resistance.
